# Long-term nitrogen fertilizer management for enhancing use efficiency and sustainable cotton (*Gossypium hirsutum* L.)

**DOI:** 10.3389/fpls.2023.1271846

**Published:** 2023-09-18

**Authors:** Yuanqi Ma, Hongchun Sun, Yurong Yang, Zhao Li, Ping Li, Yuetong Qiao, Yongjiang Zhang, Ke Zhang, Zhiying Bai, Anchang Li, Cundong Li, Liantao Liu

**Affiliations:** ^1^ State Key Laboratory of North China Crop Improvement and Regulation/Key Laboratory of North China Water-saving Agriculture, Ministry of Agriculture and Rural Affairs/Key Laboratory of Crop Growth Regulation of Hebei Province/College of Agronomy, Hebei Agricultural University, Baoding, China; ^2^ Handan Academy of Agricultural Sciences, Handan, China

**Keywords:** cotton, long-term nitrogen application, yellow river region, yield, nitrogen use efficiency

## Abstract

Optimal management of nitrogen fertilizer profoundly impacts sustainable development by influencing nitrogen use efficiency (NUE) and seed cotton yield. However, the effect of long-term gradient nitrogen application on the sandy loam soil is unclear. Therefore, we conducted an 8-year field study (2014–2021) using six nitrogen levels: 0 kg/hm^2^ (N0), 75 kg/hm^2^ (N1), 150 kg/hm^2^ (N2), 225 kg/hm^2^ (N3), 300 kg/hm^2^ (N4), and 375 kg/hm^2^ (N5). The experiment showed that 1) Although nitrogen application had insignificantly affected basic soil fertility, the soil total nitrogen (STN) content had decreased by 5.71%–19.67%, 6.67%–16.98%, and 13.64%–21.74% at 0-cm–20-cm, 20-cm–40-cm, and 40-cm–60-cm soil layers, respectively. 2) The reproductive organs of N3 plants showed the highest nitrogen accumulation and dry matter accumulation in both years. Increasing the nitrogen application rate gradually decreased the dry matter allocation ratio to the reproductive organs. 3) The boll number per unit area of N3 was the largest among all treatments in both years. On sandy loam, the most optional nitrogen rate was 190 kg/hm^2^–270 kg/hm^2^ for high seed cotton yield with minimal nitrogen loss and reduced soil environment pollution.

## 1 Introduction

Cotton (*Gossypium hirsutum* L.) is a significant cash crop in China, accounting for approximately 15% of the global planting area and 25% of output (Dai and Dong, 2014). Global cotton production involves nitrogen (N) application but has a low (30%–35%) nitrogen use efficiency (NUE) despite the currently high lint yield (Guo et al., 2020; Niu et al., 2020). N plays a decisive role in cotton growth and development because cotton demands relatively large amounts of N, which also has relatively sensitive characteristics (Iqbal et al., 2020). Surprisingly, increasing the N application rate has not increased seed cotton yield (SCY) but reduced NUE and increased input costs. This low NUE has increased environmental pollution and greenhouse gas emissions, further exacerbating the environmental burden (Sutton et al., 2011). Therefore, reducing nitrogen application and improving NUE in cotton production are crucial for achieving sustainable and stable yields (Wang et al., 2010; Zhang et al., 2017).

Furthermore, implementing scientific and reasonable tillage and management methods can prevent soil fertility degradation in poor fertilizer-retaining sandy loam soil while maintaining long-term sustainable production (Zheng et al., 2010). Boselli et al. (2020) emphasized that soil management changes soil fertility. Long-term N fertilizer application can increase the soil total nitrogen (STN) (Shiwakoti et al., 2019), nitrate, and ammonium nitrogen contents. Long-term N application experiments in Xinjiang showed that applying N to sandy loam cotton fields can effectively increase the STN, which decreases with increasing soil depth (Kaiyong et al., 2011). Initially, the nitrate nitrogen content in the soil increased, reaching a maximum in the 20-cm–40-cm soil layer, and decreased as the soil layer deepened toward the brown and Udic Haplustalf soil (in the United States Department of Agriculture system) under long-term fertilization conditions (Zhong et al., 2015).

N is crucial for crop yield and the composition factors of yield (Aulakh and Malhi, 2005; Ding et al., 2023), such as physio-ecological characteristics, dry matter (DM) accumulation, nitrogen accumulation, and NUE (Yang et al., 2013; Luo et al., 2018; Snider et al., 2021). Adequate nutrient absorption is a prerequisite for biomass accumulation and increasing SCY. However, nitrogen accumulation in the reproductive organs is crucial for high cotton economic yield (Sepaskhah and Tafteh, 2012; Khan et al., 2017b; Chen et al., 2018b). Reasonable N application effectively improves the chlorophyll content and photosynthetic rate of cotton, promoting plant photosynthesis, increasing DM accumulation, and ultimately increasing crop yield (Zhou et al., 2021). N application is crucial for crop yield, but insufficient or excessive application can negatively impact crop yield (Lin et al., 2009; Zhang et al., 2023).

N application level also significantly affected the yield composition factors (Iqbal et al., 2022). One study showed that the number of bolls per unit area and the weight of a single boll of cotton decreased when the N application rate exceeded 280 kg/hm^2^ on sandy loam (Li et al., 2019). Different N application rates significantly affect the number of bolls per unit area and the single-boll weight. N application rates exceeding 120 kg/hm^2^ did not increase the SCY on soil (Munir et al., 2015). Therefore, optimal N application is important for maximum NUE and higher lint yields (Cassman et al., 2002). Another study revealed that reducing N fertilizer application through precision tillage technology can improve wheat yield and NUE (Shah et al., 2022). Similarly, proper N reduction insignificantly affects the tiller number (Ning et al., 2022). The test time is usually 1–2 years compared to the long-term test. Therefore, the influence of the test will differ because of weather, precipitation, and other factors (Grant et al., 2016). Previous studies have demonstrated that long-term fixed N application, specifically, the 14-year fixed fertilization treatment, significantly affects soil N mineralization (Ge et al., 2018). Additionally, there were noticeable variations in the nutrients within the 0-cm–60-cm soil layer after different N application treatments, an effective indicator of the effects of different nitrogen levels on soil fertility (Chen et al., 2018a). A 16-year positioning fertilization research confirmed further that N application significantly affects soil nutrients and that proper N application can improve soil fertility, meet the nutrient requirements of crops, and increase yield (Chu et al., 2007). However, long-term N localization experiments in cotton fields with sandy loam soils are still lacking.

Therefore, we conducted a long-term N localization experiment with five nitrogen application levels over 8 years (from 2014 to 2021) to determine whether long-term N reduction can significantly reduce SCY under sandy loam soil. The study focused on the effects of different N application rates on the soil N spatial content, morphology, NUE, and yield. The aim was to provide a theoretical basis and technical support for an optimal N fertilizer application rate in cotton fields with sandy loam soil.

## 2 Materials and methods

### 2.1 Experimental sites

**Figure 1:**
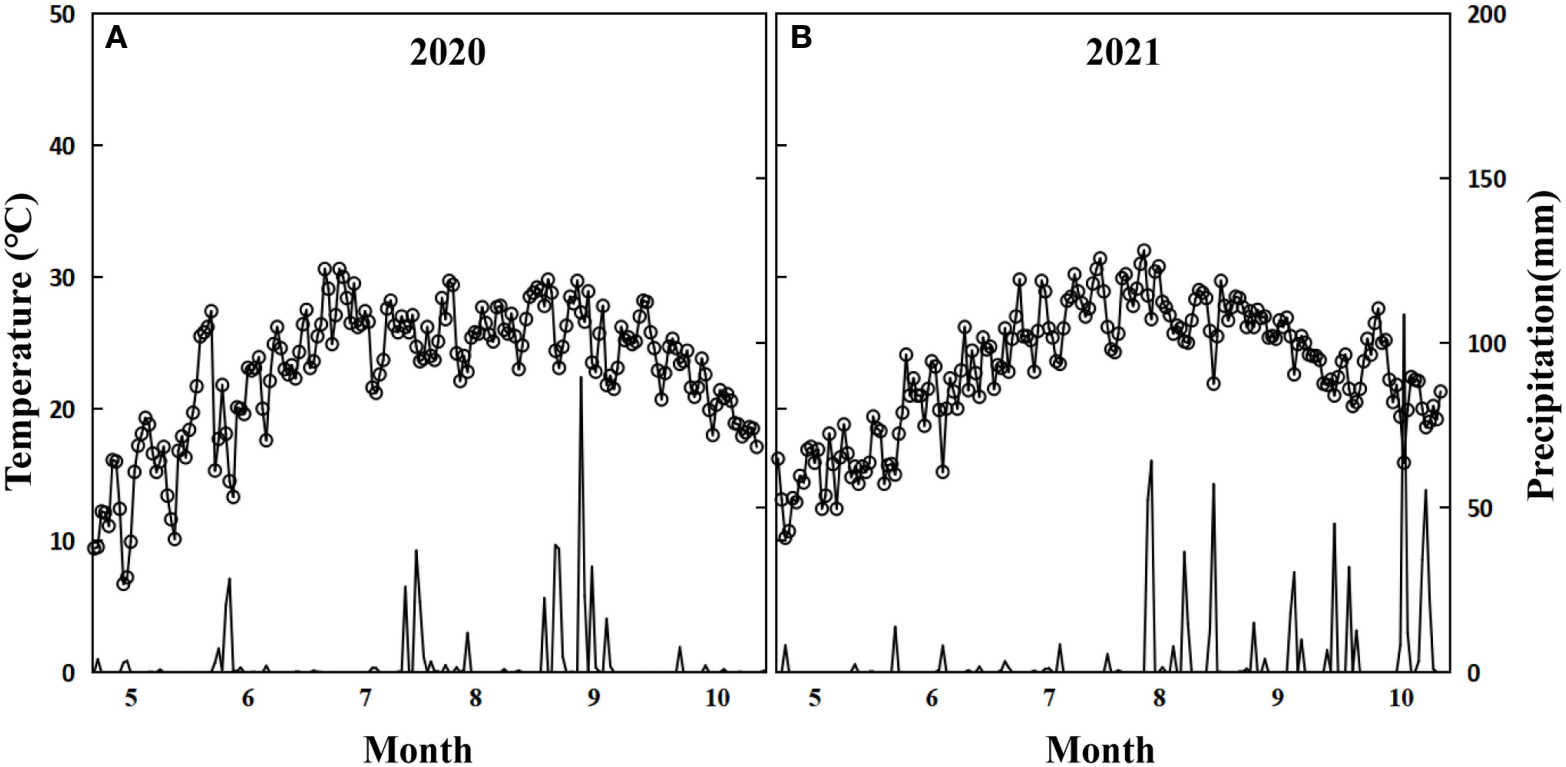
Average daily temperature and precipitation for 2020 **(A)** and 2021 **(B)**.

The experiment was conducted at the Weixian Experimental Station of the Agricultural University of Hebei (115.35°E, 37.08°N) from 2014 to 2021. The experimental field had sandy loam soil containing total nitrogen of 0.565 g·kg^-1^, available phosphorus of 5.82 mg·kg^-1^, available potassium of 112 mg·kg^-1^, and organic matter of 7.6 g·kg^-1^, with a pH of 8.1. We focused on the growth and development traits of cotton from 2020 to 2021. Basic soil fertility is shown in **Table 1**. Throughout the growing season (April–October), the average temperature and total rainfall were 21.5°C and 469.9 mm (**Figure 1A**) in 2020 and 21.7°C and 815.1 mm (**Figure 1B**) in 2021.

**Table 1:** Basic soil fertility in 2020 and 2021.

Year	Treatment	Total nitrogen	Alkali-hydrolyze nitrogen	Available phosphorus	Available potassium	Organic matter
		(g/kg)	(mg/kg)	(mg/kg)	(mg/kg)	(g/kg)
2020	N0	0.54	47.00	6.33	145.00	6.80
	N1	0.59	50.23	7.08	158.33	7.34
	N2	0.61	55.83	7.40	156.67	7.45
	N3	0.66	55.60	7.34	166.67	7.93
	N4	0.67	58.87	7.30	165.00	8.25
	N5	0.68	59.33	7.38	178.33	8.39
2021	N0	0.54	52.33	6.77	148.33	6.98
	N1	0.59	55.67	7.00	168.33	7.38
	N2	0.61	54.00	7.21	170.67	7.80
	N3	0.67	54.33	7.15	172.33	7.95
	N4	0.68	52.67	7.20	171.00	7.86
	N5	0.68	53.67	7.33	173.67	7.78

N0, N1, N2, N3, N4, and N5 represent six nitrogen rates: 0 kg/hm^2^, 75 kg/hm^2^, 150 kg/hm^2^, 225 kg/hm^2^, 300 kg/hm^2^, and 375 kg/hm^2^, respectively.

### 2.2 Experimental design

The field experiment was conducted using a randomized complete block design with six nitrogen levels: 0 kg/hm^2^, 75 kg/hm^2^, 150 kg/hm^2^, 225 kg/hm^2^, 300 kg/hm^2^, and 375 kg/hm^2^, represented by N0, N1, N2, N3, N4, and N5, respectively. Urea was the N source in 18 plots of the random blocks with three replications. The basal fertilizers were 105 kg/hm^2^ of P_2_O_5_ and 105 kg/hm^2^ of K_2_O, and urea was the N source. Nitrogen fertilizer was applied in two parts: base and topdressing fertilizer. Topdressing was performed at the full flowering stage, and the ratio of nitrogen base fertilizer to topdressing was 6:4.

The cotton cultivar, Jimian No. 863, was sown on 25th April each year at a planting density of 75,000 plants/hm^2^ (equal row spacing of 76 cm). The area of each plot was 66 m^2^ and remained unchanged during the experiment. The plants were harvested on 15th October of each year. Other field management measures, such as weeding and pest control, were conducted according to local practices.

### 2.3 Shoot morphological indicators

Six representative cotton plants were selected from each plot to measure the average of the indicators at the cotton opening stage. The plant height was measured from the first cotyledonary node to the apical bud using a ruler, and the stem diameter was measured from the first cotyledonary node using a Vernier caliper.

### 2.4 Dry matter accumulation and distribution

DM was determined at 70, 100, and 130 days after sowing (DAS). The three uniform-sized plants were separated into the root, main stem, main stem leaf, leaf branch, leaf branch leaf, leaf branch cotton boll, fruit branch, fruit branch leaf, and fruit branch cotton boll. We adopted the shovel-omics method to obtain the DM weight of the root system (length, 40 cm; width, 30 cm; height, 18 cm) (Shao et al., 2019). The separate plant sections were numbered accordingly, put in paper bags, and dried in the oven at 105°C for 30 min. After drying at 80°C to a constant weight, an electronic balance (OHAUS, NJ, USA) was used to determine the DM weight.

### 2.5 Soil total nitrogen

At five selected sites in each plot, soil samples were taken from 0-cm–20-cm, 20-cm–40-cm, and 40-cm–60-cm soil layers of each site using a soil drill at four stages (before sowing, bud stage, flower-boll stage, and withdrawal stage) in 2021. The samples were air-dried naturally, ground, sieved through a 60-mesh sieve, and digested with H_2_SO_4_-H_2_O_2_. STN content was determined using the continuous flow chemical analyzer (Auto Analyzer3, SEAL Analytical, WI, USA).

### 2.6 Nitrogen content and use efficiency

1) Nitrogen accumulation and distribution in cotton plant organs

At 70, 100, and 130 days, six uniform-sized cotton plants were randomly selected from each plot, and their organs were divided into vegetative and reproductive parts. The plants were decomposed according to the vegetative (root, main stem, main stem leaf, leaf branch, leaf branch leaf, fruit branch, fruit branch leaf) and reproductive (leaf branch cotton boll, fruit branch cotton boll) organs according to the biomass measurements. After all of the parts were dried, crushed, and sieved through a 60-mesh sieve, the DM was digested in H_2_SO_4_-H_2_O_2_, and total nitrogen content was determined by continuous flow chemical analyzer (Auto Analyzer3, SEAL Analytical, WI, USA) (Jiang et al., 2021).

2) The nitrogen use efficiency determination (Dong et al., 2020)

Agronomic nitrogen use efficiency (aNUE) = [SCY in N-application plots – SCY in N-omission plots (kg)]/The amount of applied N fertilizer (kg).

Nitrogen recovery efficiency (NRE) = (total N uptake of the N-fertilized plots − total N uptake of zero-N plots/The amount of applied N fertilizer.

Physiological nitrogen use efficiency (pNUE) = [SCY in N-application plots – SCY in N-omission plots (kg)]/[N accumulation in N-application plots – N accumulation in N-omission plots (kg)]

Internal nitrogen use efficiency (iNUE) = Lint yield (kg)/N accumulation in a plant (kg)

Partial productivity of nitrogen fertilizer (Pfpn) = SCY in N-application plots (kg)/The amount of applied N fertilizer (kg)

### 2.7 Cotton yield and component factors

On 15th October, 20 consecutive cotton plants in two rows in the middle of each plot were selected for harvesting to determine SCY. The regression analysis was conducted using 3-year values of SCY (2017–2019). The boll weight and number were determined for 50 randomly sampled bolls per plot during each harvest. Seed cotton was weighed after drying the bolls in the sun for 15 days, and the lint percentage was determined after ginning.

### 2.8 Statistical analysis

Microsoft Excel 2010 was used to record and organize the data analyzed in SPSS 26.0 (IBM Corp., Armonk, NY, USA) using the two-factor ANOVA test. The mean values of each treatment were compared at a 5% significance level (*p* ≤ 0.05) by a T-test Least Significant Difference (LSD). Correlation analysis was performed using Origin Pro 2022b (OriginLab, Northampton, MA, USA). All of the results were plotted using GraphPad Prism 8 (GraphPad Software Inc., CA, USA).

## 3 Results

### 3.1 Effects of nitrogen application on soil total nitrogen content

Soil depth and N application significantly affected STN contents at each growth stage. The STN content decreased over the growth period and soil depth (**Table 2**). The STN contents of N0, N1, N2, N3, N4, and N5 in the 20-cm–40-cm and 40-cm–60-cm soil layers decreased by 8.82%–14.30% and 30.43%–36.98%, respectively, compared to the 0-cm–20-cm soil layer. The N in each soil layer was mainly from the accumulation in the previous year, so the difference in the 20-cm–40-cm soil layer was more significant than that in the 0-cm–20-cm soil layer. At 70 DAS, the STN contents of N0, N1, N2, N3, N4, and N5 treatments in the 20-40 and 40-60 cm soil layers decreased by 2.68%–14.81% and 23.78%–33.04%, respectively, compared to the 0-cm–20-cm soil layer. At 100 DAS, the STN contents in the 20-cm–40-cm and 40-cm–60-cm soil layers decreased by 5.65%–13.53% and 20.40%–24.58%, respectively, compared to the 0-cm–20-cm soil layer. However, fertilization at 70 and 130 days decreased the differences between N2 and N3 treatments in the 0-cm–20-cm soil layer and N1 and N2 in the 20-cm–40-cm soil layer. Moreover, increasing the fertilizer application rate increased the STN contents in the two soil layers. As expected, N0 had significantly lower STN contents than other treatments. At 130 DAS, the STN contents of N0, N1, N2, N3, N4, and N5 in the 20-cm–40-cm and 40-cm–60-cm soil layers decreased by 7.40%–22.41% and 25.21%–35.01%, respectively, compared to the 0-cm–20-cm soil layer.

**Table 2:** Total nitrogen content in the 0-cm–60-cm soil layers before sowing, 70 days after sowing (DAS), 100 DAS, and 130 DAS.

Treatment	Soil layer	Before sowing	70 DAS	100 DAS	130 DAS
	(cm)	(g/kg)	(g/kg)	(g/kg)	(g/kg)
N0	0-20	0.57 ± 0.05b	0.54 ± 0.01c	0.63 ± 0.01c	0.68 ± 0.05b
	20-40	0.52 ± 0.01c	0.51 ± 0.01c	0.55 ± 0.02c	0.52 ± 0.05b
	40-60	0.40 ± 0.01c	0.41 ± 0.01b	0.50 ± 0.01b	0.47 ± 0.01b
N1	0-20	0.59 ± 0.02b	0.62 ± 0.01b	0.67 ± 0.02bc	0.68 ± 0.02b
	20-40	0.53 ± 0.03c	0.53 ± 0.01c	0.60 ± 0.02b	0.62 ± 0.01a
	40-60	0.41 ± 0.01bc	0.42 ± 0.01b	0.52 ± 0.01b	0.47 ± 0.01b
N2	0-20	0.61 ± 0.02b	0.63 ± 0.01b	0.69 ± 0.02a	0.73 ± 0.02a
	20-40	0.54 ± 0.03bc	0.58 ± 0.04b	0.62 ± 0.02ab	0.62 ± 0.02ab
	40-60	0.41 ± 0.01bc	0.43 ± 0.01b	0.52 ± 0.02b	0.48 ± 0.01b
N3	0-20	0.69 ± 0.05a	0.66 ± 0.01ab	0.70 ± 0.02a	0.74 ± 0.03a
	20-40	0.59 ± 0.01ab	0.60 ± 0.01b	0.66 ± 0.01a	0.63 ± 0.09a
	40-60	0.44 ± 0.01abc	0.48 ± 0.03a	0.53 ± 0.03ab	0.50 ± 0.03b
N4	0-20	0.70 ± 0.02a	0.66 ± 0.03a	0.72 ± 0.01a	0.74 ± 0.02a
	20-40	0.60 ± 0.03a	0.64 ± 0.02a	0.67 ± 0.01a	0.64 ± 0.02a
	40-60	0.44 ± 0.01ab	0.50 ± 0.03a	0.54 ± 0.02ab	0.51 ± 0.03b
N5	0-20	0.68 ± 0.01a	0.69 ± 0.03a	0.72 ± 0.01a	0.75 ± 0.01a
	20-40	0.60 ± 0.04a	0.65 ± 0.02a	0.67 ± 0.05a	0.67 ± 0.05a
	40-60	0.46 ± 0.04a	0.51 ± 0.03a	0.58 ± 0.03a	0.56 ± 0.03a
Source of variation					
Soil layer		*	*	*	*
N rate		*	*	*	*
S×N		ns	ns	ns	ns

N0, N1, N2, N3, N4, and N5 represent six nitrogen treatments: 0 kg/hm^2^, 75 kg/hm^2^, 150 kg/hm^2^, 225 kg/hm^2^, 300 kg/hm^2^, and 375 kg/hm^2^, respectively. Values are means ± standard error (n = 5). Different lowercase letters on the rows indicate significant differences between treatments (p ≤ 0.05).

*Significant at 95% confidence level. ns means no significance at 95% confidence level.

DAS, days after sowing.

### 3.2 Effects of nitrogen application on cotton plant morphological characteristics

Different N application rates significantly affected the morphology of cotton plants (**Table 3**). For instance, increasing the N application level for treatments N1 and N2 increased the plant height by 7.07% and 14.72%, respectively, compared with N0. N3 resulted in the highest stem diameter, which increased by 33.61%, 12.26%, 7.21%, 9.17%, and 14.42% compared to N0, N1, N2, N4, and N5, respectively. Furthermore, the N3 generated the highest number of fruit branches, with 56.25%, 4.17%, 31.58%, 19.05%, and 4.17% increments compared to N0, N1, N2, N4, and N5. However, there was no significant difference in the plant height of the N3 and N5 treatments. The N0 treatment significantly reduced the number of fruit branches compared to N1, N2, N4, and N5.

**Table 3:** Effects of different nitrogen treatments on plant morphological indexes.

Treatment	Height (cm)	Stem diameter (cm)	Fruit branches
N0	69.3 ± 2.2c	7.9 ± 0.2d	8.0 ± 0.0d
N1	74.2 ± 5.4c	10.6 ± 0.1c	12.0 ± 0.8ab
N2	78.2 ± 3.8bc	11.1 ± 0.2ab	9.5 ± 0.4cd
N3	79.5 ± 5.2a	11.9 ± 0.5a	12.5 ± 0.4a
N4	77.5 ± 2.7ab	10.9 ± 0.2b	10.5 ± 0.4bc
N5	84.0 ± 4.9a	10.4 ± 0.6ab	12.0 ± 1.6ab

N0, N1, N2, N3, N4, and N5 represent six nitrogen treatments: 0 kg/hm^2^, 75 kg/hm^2^, 150 kg/hm^2^, 225 kg/hm^2^, 300 kg/hm^2^, and 375 kg/hm^2^, respectively. Values are means ± standard error (n = 3). Different lowercase letters on the rows indicate significant differences between treatments (p ≤ 0.05).

### 3.3 Effects of gradient nitrogen application on nitrogen use efficiency

The year of planting did not significantly affect the NUE. Additionally, N rates significantly affect iNUE, NRE, aNUE, and Pfpn (*p* ≤ 0.05). The interaction between year and N rate significantly influenced NRE and aNUE (*p* ≤ 0.05). Compared with 2020, iNUE, NRE, aNUE, pNUE, and Pfpn decreased in 2021 under the same nitrogen application treatment. The NRE decreased significantly with the increase in N application (**Table 4**). In 2020, increasing the N application rate significantly decreased the aNUE, iNUE, and partial nitrogen productivity (Pfpn). The iNUE of N1–N5 treatments decreased by 3.73%, 13.88%, 18.81%, 17.61%, and 18.81%, respectively, compared to the N0 treatment. Additionally, the NRE of N2–N5 treatments decreased by 10.57%, 25.36%, 44.24%, and 45.09%, respectively, compared with the N1 treatment. The aNUE and pNUE also decreased by 45.22%, 61.26%, 74.39%, and 82.01%, and 38.58%, 49.00%, 53.92%, and 60.32% at N1–N5, respectively, compared with N1. Furthermore, Pfpn decreased by 49.41%, 66.02%, 74.92%, and 80.23% at N1–N5, respectively, compared with N1 (**Table 4**). The trend was similar in 2021, indicating that higher N application rates decrease iNUE and Pfpn. However, increasing the nitrogen application rate initially increased the aNUE and pNUE before decreasing.

**Table 4:** Effects of different nitrogen treatments on the nitrogen use efficiency of cotton.

Year	N rate	iNUE	NRE	aNUE	pNUE	>Pfpn
		(kg/kg)	(%)	(kg/kg)	(kg/kg)	(kg/kg)
2020	N0	6.70 ± 0.25a				
	N1	6.45 ± 0.33a	39.74 ± 3.60a	6.17 ± 2.29a	15.45 ± 5.10a	51.35 ± 2.29a
	N2	5.77 ± 0.31b	35.54 ± 0.29a	3.38 ± 1.52ab	9.49 ± 4.21ab	25.98 ± 1.52b
	N3	5.44 ± 0.07b	29.66 ± 2.41b	2.39 ± 0.88b	7.88 ± 2.34b	17.45 ± 0.88c
	N4	5.52 ± 0.08b	22.16 ± 0.61c	1.58 ± 0.21b	7.12 ± 0.74b	12.88 ± 0.21d
	N5	5.44 ± 0.15b	17.92 ± 1.85c	1.11 ± 0.28b	6.13 ± 1.11b	10.15 ± 0.28d
2021	N0	6.57 ± 0.19a				
	N1	5.79 ± 0.14b	37.06 ± 0.78a	2.27 ± 1.04ab	6.19 ± 2.87a	48.48 ± 1.04a
	N2	5.61 ± 0.25b	34.71 ± 0.60b	3.22 ± 1.41a	9.34 ± 4.20a	26.32 ± 1.41b
	N3	5.38 ± 0.23bc	33.28 ± 0.25b	2.62 ± 0.41ab	7.88 ± 1.25a	18.02 ± 0.41c
	N4	4.90 ± 0.21cd	24.69 ± 0.87c	1.22 ± 0.59ab	5.02 ± 2.52a	12.77 ± 0.59d
	N5	5.04 ± 0.09d	19.85 ± 0.40d	1.07 ± 0.08b	5.38 ± 0.48a	10.31 ± 0.08e
Source of variation						
Year		ns	ns	ns	ns	ns
N rate		*	*	*	ns	*
Y×N		ns	*	*	ns	ns

N rate, nitrogen rate; iNUE, internal nitrogen use efficiency; NRE, nitrogen recovery efficiency; aNUE, agronomic nitrogen use efficiency; pNUE, physiological nitrogen use efficiency; Pfpn, partial productivity of nitrogen fertilizer. Different lowercase letters on the row indicate significant differences between treatments (p ≤ 0.05). N0, N1, N2, N3, N4, and N5 represent six nitrogen treatments: 0 kg/hm^2^, 75 kg/hm^2^, 150 kg/hm^2^, 225 kg/hm^2^, 300 kg/hm^2^, and 375 kg/hm^2^, respectively. Values are means ± standard error (n = 3).

*Significant at 95% confidence level. ns means no significance at 95% confidence level.

N rate, nitrogen rate; iNUE, internal nitrogen use efficiency; NRE, nitrogen recovery efficiency; aNUE, agronomic nitrogen use efficiency; pNUE, physiological nitrogen use efficiency; Pfpn, partial productivity of nitrogen fertilizer.

### 3.4 Effects of nitrogen application on dry matter accumulation, nitrogen accumulation, and yield components of cotton

#### 3.4.1 Dry matter and nitrogen accumulation of the cotton plant

The N rate and year significantly affected DM accumulation, DM accumulation of reproduction organs, nitrogen accumulation, and nitrogen accumulation of reproduction organs (*p* ≤ 0.05). The interaction of year and N rate significantly influenced DM accumulation, DM accumulation of reproduction organs, and nitrogen accumulation (*p* ≤ 0.05). Enhancing N application resulted in higher DM and N accumulation in 2021 than those in 2020. Different N application rates significantly affected the total DM accumulation (**Table 5**). Specifically, N5 and N3 treatments exhibited the highest DM accumulation in 2020 and 2021, increasing by 19.89% and 22.37%, respectively, compared to the N0 treatment. In 2020, N1–N5 treatments increased the DM accumulation of the vegetative organs and reproduction organs by 17.14%–30.81% and 2.92%–9.58%, respectively.

**Table 5:** Effects of different nitrogen treatments on dry matter accumulation and nitrogen accumulation in cotton plants.

Year	Treatment	Dry matter accumulation	Dry matter accumulation of reproduction organs	Nitrogen accumulation	Nitrogen accumulation of reproduction organs
		(kg/hm^2^)	(kg/hm^2^)	(kg/hm^2^)	(kg/hm^2^)
2020	N0	10557 ± 34.49d	5151 ± 7.27d (48.79)	211.68 ± 1.41d	140.49 ± 0.65d (66.37)
	N1	11635 ± 90.07c	5303 ± 12.25c (45.58)	241.48 ± 2.70c	150.32 ± 1.29c (62.25)
	N2	12168 ± 51.58b	5546 ± 5.70b (45.58)	264.99 ± 0.44b	158.05 ± 1.34b (59.64)
	N3	12600 ± 186.39a	5644 ± 60.03a (44.80)	278.41 ± 5.42a	161.48 ± 2.78a (58.00)
	N4	12539 ± 104.55a	5628 ± 66.92ab (44.88)	278.17 ± 1.84a	161.11 ± 2.73a (57.92)
	N5	12657 ± 149.64a	5585 ± 35.20ab (44.13)	278.88 ± 6.93a	160.07 ± 2.30a (57.40)
2021	N0	10517 ± 28.20d	5240 ± 20.39d (49.82)	213.98 ± 0.72d	142.70 ± 0.36d (66.69)
	N1	11484 ± 12.96c	5538 ± 12.22c (48.23)	241.78 ± 0.58c	153.48 ± 0.38c (63.48)
	N2	12176 ± 43.05b	5762 ± 16.45b (47.33)	266.05 ± 0.91b	161.57 ± 0.32b (60.73)
	N3	12870 ± 34.52a	5864 ± 8.52a (45.56)	288.87 ± 0.56a	167.62 ± 0.76a (58.03)
	N4	12828 ± 99.71a	5850 ± 14.72a (45.60)	288.04 ± 2.60a	167.58 ± 0.60a (58.18)
	N5	12864 ± 66.29a	5884 ± 6.37a (45.74)	288.40 ± 1.50a	167.58 ± 1.18a (58.10)
Source of variation					
Year		*	*	*	*
N rate		*	*	*	*
Y×N		*	*	*	ns

The values within brackets indicate the ratios of dry matter accumulation and nitrogen accumulation of the vegetative organs to the total dry matter accumulation and total nitrogen accumulation. Different lowercase letters on the rows indicate significant differences between treatments (p ≤ 0.05). N0, N1, N2, N3, N4, and N5 represent six nitrogen treatments: 0 kg/hm^2^, 75 kg/hm^2^, 150 kg/hm^2^, 225 kg/hm^2^, 300 kg/hm^2^, and 375 kg/hm^2^, respectively. Values are means ± standard error (n = 3).

*Significant at 95% confidence level. ns means no significance at 95% confidence level.

During the opening stage, the N5 treatment generated the maximum DM accumulation in the vegetative organs, although the amount was not significantly different from N3 and N4 treatments. The maximum DM accumulation of reproduction organs was observed under the N3 treatment, although the value was not significantly different from N4 and N5 treatments. In 2021, N1–N5 treatments increased the DM accumulation of vegetative organs and reproduction organs by 12.67%–32.76% and 5.7%–11.01%, respectively, compared to N0. The DM accumulation of vegetative organs peaked under the N3 treatment, although this peak was not significantly different from N4 and N5 treatments. Meanwhile, the reproduction organs reached their maximum (5,884 kg/hm^2^) under the N5 treatment, which was not significantly different from the N3 and N4 treatments. The DM accumulation of N0 was significantly lower than all of the other treatments.

#### 3.4.2 Yield components

The N rate and year significantly affected the boll number per unit area, boll weight, and lint percentage (*p* ≤ 0.05). Moreover, the N rate also significantly affected SCY. However, the interaction between N and year had no significant effect on SCY and its constituent factors (*p* ≤ 0.05). Different N application rates significantly affected the SCY, boll number per unit area, boll weight, and lint percentage (**Table 6**). Treatment N3 caused the highest SCY over the 2 years. In 2020, the SCY of N0 was significantly lower than the other treatments. The SCY of N1–N5 treatments increased by 13.65%, 14.98%, 15.84%, 14.02%, and 12.28%, respectively, compared to the N0 treatment. In 2021, there was no significant difference between N0 and N1 treatments, but both were significantly lower than the other treatments.

**Table 6:** Effects of different nitrogen treatments on cotton field yield and its related components.

Year	Treatment	Seed cotton yield	Number of bolls	Boll weight	Lint percentage
		(kg/hm^2^)	(×10^4^/hm^2^)	(g)	(%)
2020	N0	3388.77 ± 89.06b	70.20 ± 1.63c	4.68 ± 0.06c	41.84 ± 1.12a
	N1	3851.17 ± 172.09a	73.12 ± 0.28b	4.87 ± 0.21bc	40.40 ± 0.43ab
	N2	3896.49 ± 227.60a	75.67 ± 1.99ab	5.30 ± 0.07a	39.24 ± 0.48bc
	N3	3925.44 ± 196.90a	77.66 ± 0.52a	5.03 ± 0.09b	38.60 ± 0.79c
	N4	3863.74 ± 61.78a	77.61 ± 1.46a	5.12 ± 0.06ab	39.75 ± 0.64bc
	N5	3804.77 ± 105.84a	77.30 ± 0.81a	5.08 ± 0.18ab	39.90 ± 0.70bc
2021	N0	3465.0 ± 94.759c	61.25 ± 1.27c	5.24 ± 0.08b	40.55 ± 0.18a
	N1	3635.66 ± 77.67bc	64.50 ± 0.17b	5.87 ± 0.18a	38.47 ± 0.54b
	N2	3947.57 ± 212.21a	67.10 ± 1.21ab	5.86 ± 0.05a	37.86 ± 0.71bc
	N3	4054.78 ± 91.18a	70.45 ± 1.96a	5.75 ± 0.04a	38.32 ± 0.76b
	N4	3830.60 ± 175.85ab	68.89 ± 1.97a	5.89 ± 0.16a	36.89 ± 0.56c
	N5	3864.72 ± 30.04ab	68.10 ± 1.29a	5.67 ± 0.07a	37.61 ± 0.55bc
Source of variation					
Year		ns	*	*	*
N rate		*	*	*	*
Y×N		ns	ns	ns	ns

Different lowercase letters on the rows indicate significant treatment differences (p ≤ 0.05). N0, N1, N2, N3, N4, and N5 represent six nitrogen treatments: 0 kg/hm^2^, 75 kg/hm^2^, 150 kg/hm^2^, 225 kg/hm^2^, 300 kg/hm^2^, and 375 kg/hm^2^, respectively. Values are means ± standard error (n = 3).

*Significant at 95% confidence level. ns means no significance at 95% confidence level.

Increasing the nitrogen application rate from N1 to N5 increased the SCY by 4.92%, 13.92%, 17.02%, 10.55%, and 11.53%, respectively. However, the same increments of N application gradually decreased the DM accumulation of the reproduction organs over the 2 years. In 2020, the proportion of reproduction organs under N1–N5 treatments decreased by 3.21%, 3.21%, 3.99%, 3.91%, and 4.66%, respectively, compared with N0. Similarly, the proportion of reproduction organs under N1–N5 treatment decreased by 1.59%, 2.49%, 4.26%, 4.22%, and 4.08%, respectively, in 2021. There was no significant difference between N3, N4, and N5 treatments (*p* ≤ 0.05).

The boll number per unit area fluctuated with increasing N application rate. In 2020 and 2021, N3 treatment generated the highest boll number per unit area. Boll number per unit area increased by 14.08%, 25.18%, 31.52%, 31.41%, and 31.75% under N1–N5 treatments in 2020. The boll number per unit area increased with an increasing N application rate (N1–N5), ranging from 12.99% to 34.78% in 2021. However, the boll weight was significantly lower under N0 than the other treatments. In 2020 and 2021, the boll weight increased with each level of N treatment, the highest occurring under N2 treatment in 2020 (13.25%) and N1 treatment in 2021 (12.02%). However, increasing the N application rate significantly decreased the lint percentage.

**Figure 2:**
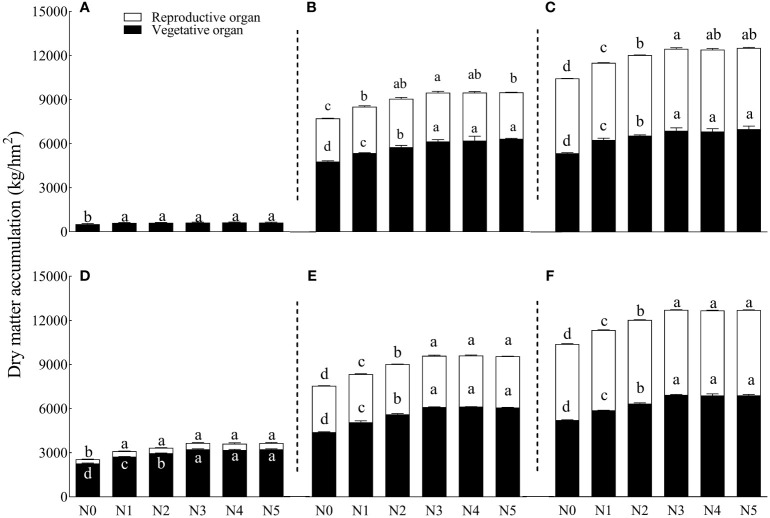
Effects of different nitrogen gradients on dry matter accumulation in vegetative and reproductive organs of cotton at different growth stages. Where 70 DAS, 100 DAS, and 130 DAS are represented by panels **(A–C)** for 2020 and by panels **(D–F)** for 2021, respectively. DAS, days after flowering. Different lowercase letters on the row indicate significant differences between treatments (*p* ≤ 0.05). N0, N1, N2, N3, N4, and N5 represent six nitrogen treatments: 0 kg/hm^2^, 75 kg/hm^2^, 150 kg/hm^2^, 225 kg/hm^2^, 300 kg/hm^2^, and 375 kg/hm^2^, respectively. Error bars are the standard errors of the means. DAS, days after sowing.

**Figure 3:**
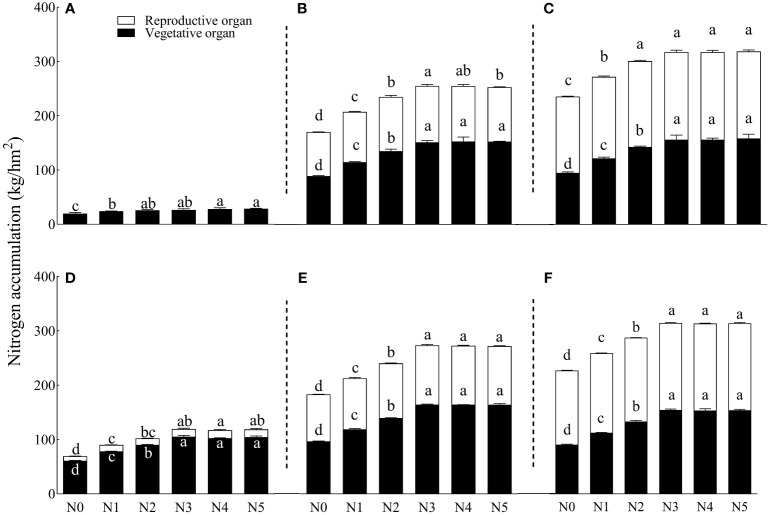
Effects of different nitrogen gradients on nitrogen accumulation in the vegetative and reproductive organs of cotton at different growth stages. Where 70 DAS, 100 DAS, and 130 DAS are represented by panels **(A–C)** for 2020 and by panels **(D–F)** for 2021, respectively. DAS, days after flowering. Different lowercase letters on the rows indicate significant treatment differences (*p* ≤ 0.05). N0, N1, N2, N3, N4, and N5 represent six nitrogen treatments: 0 kg/hm^2^, 75 kg/hm^2^, 150 kg/hm^2^, 225 kg/hm^2^, 300 kg/hm^2^, and 375 kg/hm^2^, respectively. Error bars are the standard errors of the means. DAS, days after sowing.

Different N application rates significantly affected nitrogen accumulation and nitrogen accumulation of the reproduction organs (**Figure 2**). During the opening stages in 2020 and 2021, treatments N5 and N3 caused the maximum nitrogen accumulation (31.75% and 35%, respectively) compared to N0. In 2020, each N treatment increased nitrogen accumulation of the vegetative organs and reproduction organs by 17.14%–30.81% and 2.92%–9.58%, respectively, compared to N0. At the opening stage, nitrogen accumulation of vegetative organs peaked under the N5 treatment, but that peak was not significantly different from the N3 and N4 treatments (**Figure 3**). Treatment N3 yielded the highest nitrogen accumulation of the reproduction organs, although the accumulation was not significantly different from N2, N4, and N5 treatments.

In 2021, each N treatment increased nitrogen accumulation of vegetative organs and reproduction organs by 12.67%–32.76% and 5.7%–11.01%, respectively, compared to the N0 treatment. Under the N3 treatment, nitrogen accumulation of vegetative organs peaked during the opening stage, but that peak was not significantly different from N4 and N5 treatments. Treatment N5 caused the highest nitrogen accumulation of reproduction organs. Treatment N0 accumulated the least N, significantly lower than the other N treatments.

#### 3.4.3 Unary linear regression analysis of the effects of nitrogen application rate on cotton seed cotton yield

Applying 193.59 kg/hm^2^, 269.72 kg/hm^2^, 214.35 kg/hm^2^, 230.77 kg/hm^2^, and 309.22 kg/hm^2^ of N fertilizer caused maximum yields in 2017, 2018, 2019, 2020, and 2021, respectively (**Table 7**). The test site had sandy loam soil and received heavy rainfall during the 2021 growth period. This heavy rainfall caused soil N loss, increasing the N needed for maximum SCY. Therefore, the recommended N application range for sustainable cotton production in this area is 190 kg/hm^2^–270 kg/hm^2^. In rainy years, higher N input is required to ensure optimal yields.

**Table 7:** Regression analysis of the effect of different nitrogen treatments on cotton seed yield over 5 years.

Year	Regression equation	R^2^	Theoretical optimal nitrogen application rate (kg/hm^2^)
2017	y = -0.0173x^2^ + 6.6987x + 3648.0	0.9437*	193.59
2018	y = -0.0107x^2^ + 5.7722x + 3770.8	0.8937	269.72
2019	y = -0.0086x^2^ + 3.6869x + 3555.9	0.6469	214.35
2020	y = -0.0096x^2^ + 4.4308x + 3454.4	0.8772	230.77
2021	y = -0.0067x^2^ + 4.1436x + 3435.7	0.9724*	309.22

R^2^, determination coefficient; *Significant at 95% confidence level.

### 3.5 Correlation analysis

**Figure 4:**
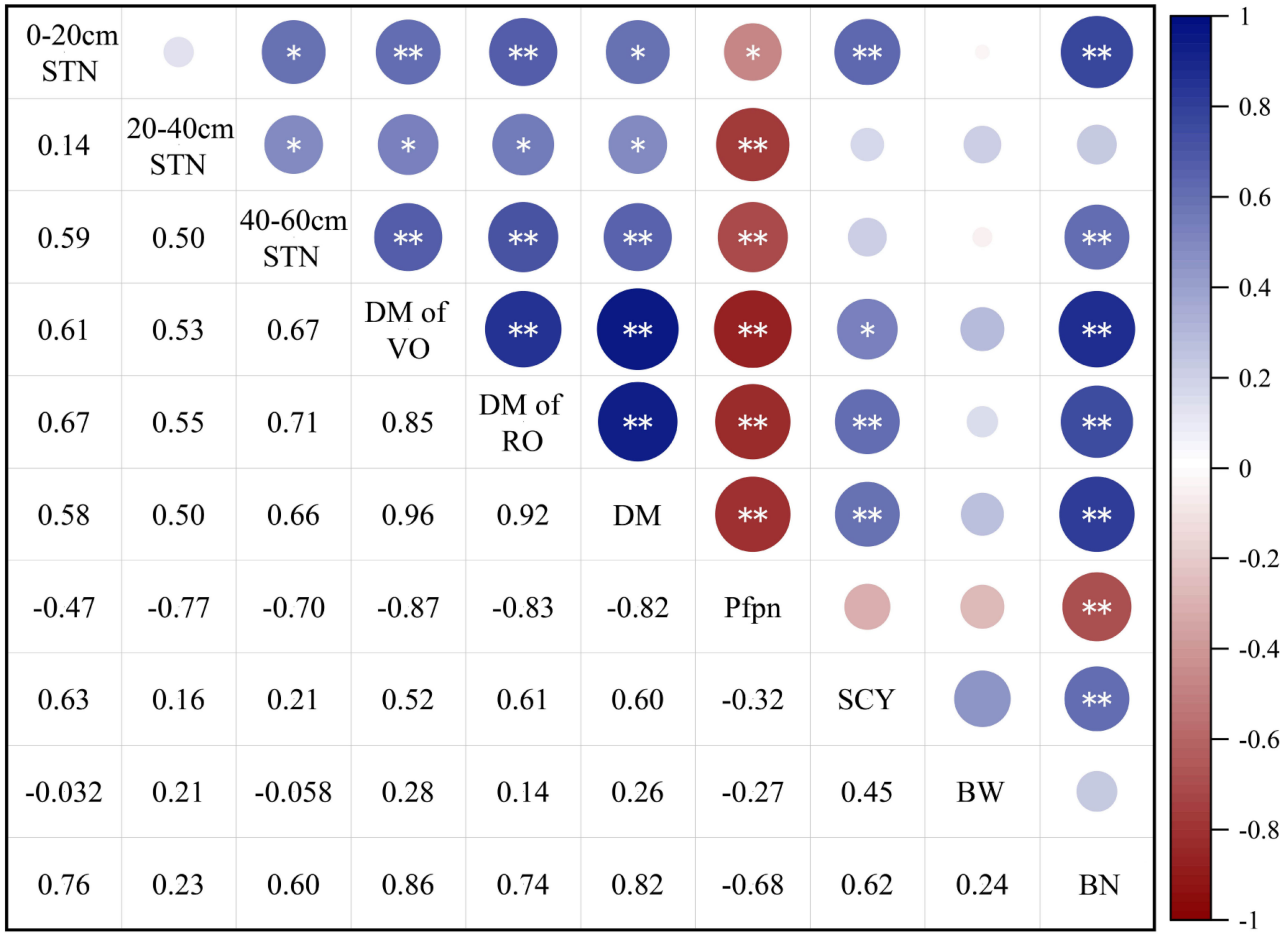
Correlation analysis of cotton growth and nitrogen utilization index under different nitrogen application rates. Numbers 1–10 represent the total nitrogen content in 0-cm–20-cm STN, 20-cm–40-cm STN, 40-cm–60-cm Soil total nitrogen (STN), dry matter of vegetative organs (DM of VO), dry matter of reproductive organs (DM of RO), DM, Pfpn, seed cotton yield (SCY), single boll weight (BW), and boll number per unit area (BN), and boll number per unit area (BN), respectively. *Significant at 95% confidence level. **Highly significant at 99% confidence level.

The STN content in the 0-cm–20-cm and 20-cm–40-cm soil layers significantly positively correlated with DM accumulation, DM accumulation of vegetative organs, DM accumulation of reproduction organs, SCY, and boll number per unit area (**Figure 4**). However, other indices negatively correlate with Pfpn. A significant positive correlation exists between SCY and the STN content in the 0-cm–20-cm soil layer. The boll number per unit area positively correlates with the STN content in the 0-cm–20-cm soil layer. However, there was a negative correlation between the boll number per unit area and iNUE. The iNUE is significant and negatively correlated with the STN content in the 0-cm–20-cm and 20-cm–40-cm soil layers.

## 4 Discussion

### 4.1 Nitrogen accumulation in cotton fields

Accumulation of STN is long-term; thus, this study examined the effects of multiple years of fixed-point gradient N application on nitrogen accumulation in cotton fields. From 2017 to 2020, N application increased the total nitrogen and alkali-hydrolyzed nitrogen contents in the 0-cm–60-cm soil layers of each treatment. At 130 DAS, the STN content of the 0-cm–60-cm soil layer increased by 0.84% to 22.40% compared to the STN content before sowing. The STN content decreased with increasing soil depth (**Table 2**). Moreover, the STN content was higher in the 0-cm–40-cm than in the 40-cm–60-cm soil layer. The STN content was significantly lower in the N0 than in the other treatments. In contrast, the total nitrogen content was not significantly different between 225-kg/hm^2^ and 375-kg/hm^2^ N application treatments. Nonetheless, the soil nitrogen fertility level of each treatment remained low due to the poor fertilizer-retention characteristic of sandy loam soil. This study suggests that sole long-term N fertilizer application cannot maintain the soil nutrient levels (Su et al., 2006); hence, long-term single N application in loam soil has minimal effects on the STN content (Hai et al., 2010).

Various factors influence the soil environment, including microorganisms, precipitation, nutrient absorption, and crop utilization. The STN content in the 0-cm–20-cm layer significantly differed from the other layer. As the soil depth increased, the STN content of the 40-cm–60-cm soil differed significantly only among individual treatments because N accumulates in the soil profile slowly (Yang et al., 2011b). Moreover, increasing the soil depth decreased the STN content. After 19 years of continuous N application in loam soil, Duan et al. (2016) obtained similar results on the STN content in the 0-cm–40-cm soil layer. However, no significant difference was observed in the STN content beyond a soil depth of 40 cm, consistent with a previous report (Emiru and Gebrekidan, 2013). Moreover, the STN content in the 40-cm–100-cm soil layer significantly increased after 19 years of continuous N application, suggesting that excessive N application can cause the unabsorbed N by plants to enter the deeper soil layers. In contrast, residual N moves deeper into the soil beyond the root system, leading to N loss (Liu et al., 2017). This process occurs gradually and is challenging to detect. Therefore, reasonable N application rates are recommended to ensure optimal crop yield and prevent N loss (Ju et al., 2009).

Low N application rates cause soil N depletion. However, N application rates that exceed the crop demand lead to excess N in the soil or N loss (Fageria and Baligar, 2005). This study showed that the STN content was significantly higher in the eighth year after continuous fixed-point application of 225-kg/hm^2^–375-kg/hm^2^ N than in the N0 treatment. The 13-year experiment by Tian et al. (2018) showed that nitrogen accumulation was significantly increased in the 0-cm–20-cm soil layer at 180-kg/hm^2^ nitrogen application rate compared with long-term no-nitrogen treatment. The 17-year fertilizer trials by Tong et al. (2009) involving eight study sites also showed that long-term rational nitrogen application could increase the STN content in plow layers. Moreover, the experiment by Meng et al. (2005) also demonstrated that long-term fixed point quantitative nitrogen application could significantly increase STN content at 150-kg/hm^2^ nitrogen application rate. These results are similar to the long-term results of Aula et al. (2016) on sandy loam soil, proving that increasing the N application rate increases STN content.

### 4.2 Dry matter and nitrogen accumulation and utilization by cotton plants

Nitrogen is among the most important elements affecting plant growth (Dubey et al., 2021). Insufficient or excessive N application hinders DM accumulation in cotton (Boquet and Breitenbeck, 2000). This study showed that DM accumulated most with 225 kg/hm^2^ of N application in 2020 and 2021, improving the plant height, stem diameter, and fruit branch number. The DM decreased with the decreased or increased N application. Very low nitrogen levels probably caused nitrogen deficiency in cotton, and plants were unable to grow normally, thus affecting plant DM accumulation and growth and development of reproductive organs. However, higher levels caused excessive vegetative growth, inconducive for the growth and development of cotton bolls in the later period, hence, low DM. Therefore, non-optimal N application rates do not increase DM accumulation but decrease DM accumulation in cotton plants (Yang et al., 2011a). Wang et al. (2021) showed similar results, that ≤200 kg/hm^2^ of N decreased the DM accumulation of all organs.

Increasing the N application rate increased N accumulation (Geng et al., 2015; Wang et al., 2022). This study revealed a positive relationship between N and DM accumulation and distribution during different growth stages of cotton. DM accumulation of reproduction organs, nitrogen accumulation, and nitrogen accumulation of reproduction organs were higher in 2021 than those in 2020. This is due to the difference in DM accumulation and nitrogen accumulation caused by different precipitation in the 2 years. The precipitation difference between seasons led to different DM accumulation and nitrogen accumulation and the difference in reproduction organs growth under the same N application (Mullins and Burmester, 1990). This study also showed that cotton plants that received little or no N fertilizer for an extended period exhibited weak growth and insufficient nutrients, weakening the reproductive growth. The reproduction organs are the primary nitrogen-distributing organs in the flower-boll and withdrawal stages, accounting for >50% of the distribution. Therefore, plants that did not receive N fertilizer for a year accumulated significantly lower N than N-fertilized plants. Therefore, appropriately increasing the N application rate can effectively improve the total DM accumulation in cotton plants and the reproduction organs, resulting in a higher cotton yield. However, excessive N application can inhibit the transport of photosynthetic products to the reproduction organs, negatively impacting the accumulation of nutrient elements in cotton plants and hindering plant growth and N absorption (Fritschi et al., 2003). The early growth stage is characterized by vigorous vegetative growth, but the period from flowering to flopping is crucial for DM accumulation. During this flowering to opening stage, cotton plants have a high nutrient demand, and reproductive growth is more crucial than vegetative growth (Chen et al., 2010). Thus, reproductive organs become the primary DM accumulation distribution organs. Similarly, this experiment demonstrated that cotton plants treated with 300 kg/hm^2^ of N accumulated more DM accumulation, with higher DM accumulation allocations to the reproduction organs. Increasing the N application rate to 450 kg/hm^2^ caused the largest DM accumulation, but a low proportion was distributed to the reproduction organs, unbalancing the source–sink relationship (Ma et al., 2013). Hence, a lack of N suppresses cotton growth and development, and excessive N also inhibits the reproductive growth of cotton (Leghari et al., 2016).

N application level impacted the NUE of cotton fields by decreasing NUE with increasing N application levels. Increasing the N application rate increased the recovery rate and internal utilization rate and significantly decreased the partial productivity of N (Zhang et al., 2012). Additionally, higher N application rates decreased the aNUE and pNUE in 2020. In 2021, aNUE and pNUE increased to the maximum (150 kg/hm^2^) and then decreased with increasing N application rate. This difference might have been due to the difference in precipitation, which caused N nitrification and ammonia volatilization, thus reducing NUE in 2021 compared with that in 2020. Studies have shown that crops absorb only 25%–50% of the applied N, indicating relatively low NUE (Chien et al., 2016). Relevant research showed that excessive N fertilization leads to low NUE and economic losses. However, optimizing the amount of nitrogen increased the soil nutrient content, soil catalase, urease, acid phosphatase, and invertase activities, generating a coarse soil texture (Malhi et al., 2010; Chen et al., 2021). Therefore, optimizing the N application rates based on regional production conditions, yield, and N absorption and utilization is crucial for reducing soil pollution, maintaining crop yield, improving NUE, and sustainable farmland development (Stamatiadis et al., 2016).

### 4.3 Yield components

Adequate nitrogen fertilizer application is an important prerequisite for good yield formation (Khan et al., 2017a). The experiment demonstrated that prolonged periods of no N application or low application rates significantly reduced cotton yield. The SCY and yield components were the lowest at 0-kg/hm^2^ N application rate. Appropriate N application enhanced SCY. However, excessive N application over an extended period did not continually increase the SCY, resulting in fertilizer wastage. Increasing the N application rate initially increased the boll number per unit area of cotton. The number of bolls per unit area reached the maximum under the 225-kg/hm^2^ nitrogen rate in 2020 and 2021. Other nitrogen rates decreased the same because no or low nitrogen application treatment implies insufficient nitrogen supply for the development of reproductive organs. In contrast, excessive nitrogen application promotes vegetative growth, inconducive for high yield and other yield-constituent factors. Additionally, increasing the N application rate decreased the lint fraction, suggesting that lack of or reduced N application rates reduce cotton yield and yield components. The results of this study are consistent with previous research (Devkota et al., 2013; Zonta et al., 2016; Li et al., 2017)

Multiple factors, including climate, soil, and cultivated varieties, determine the appropriate N application rate for cotton (Constable and Bange, 2015). For instance, 0 kg/hm^2^–360 kg/hm^2^ of N application increases the SCY (Min et al., 2014). In loam tidal soils, 360 kg/hm^2^ of N fertilization caused the highest SCY (with an STN content of 4 over 3-year experimentation) (Pengcheng et al., 2015). Furthermore, applying 225 kg/hm^2^–300 kg/hm^2^ of N to medium- and superior-fertility soil increased yield, but 375 kg/hm^2^ decreased yield (Li et al., 2010). Meanwhile, Xue et al. (2006) showed that 360 kg/hm^2^ achieved an optimal yield in the Anyang experiment site characterized by low STN content and 240 kg/hm^2^ in the high-fertility Nanjing site with clay soil. A regression analysis of the 5-year experimental results established that 190 kg/hm^2^ and 270 kg/hm^2^ are the optimal N application rates for sustainable cotton production in these regions.

## 5 Conclusions

The results of the 2-year field experiment based on the 6-year long-term positioning experiment showed that N application greatly affected the N content in sandy loam cotton fields. The STN content decreased, and the difference between the soil layers weakened with increasing soil depth (0 cm–20 cm, 40 cm–60 cm, 40 cm–60 cm). The STN content did not significantly increase at 300-kg/hm^2^ and 375-kg/hm^2^ N application rates. Additionally, the N absorption by cotton plants increased with increased N application. However, it decreased the NUE in cotton fields. When the N application rate exceeded 225 kg/hm^2^, the DM accumulation and nitrogen accumulation declined. Planting without N fertilizer or insufficient N application weakens the reproductive growth of cotton plants and greatly reduces the boll number per unit area and boll weight. Therefore, the suitable range of nitrogen fertilization for cotton in the sandy loam soil of the Yellow River Basin is 190 kg/hm^2^–270 kg/hm^2^. This study provides a basis for rational N application in cotton fields under sandy loam soil in the Yellow River Basin, providing theoretical support for NUE and improving ecological environments in cotton fields.

## Data availability statement

The raw data supporting the conclusions of this article will be made available by the authors, without undue reservation.

## Author contributions

YM: Data curation, Formal Analysis, Investigation, Methodology, Writing – original draft. HS: Writing – original draft, Data curation, Formal Analysis, Investigation. YY: Writing – original draft, Software, Data curation, Investigation, Methodology. ZL: Data curation, Investigation, Methodology, Writing – original draft. PL: Data curation, Investigation, Writing – original draft. YQ: Data curation, Formal Analysis, Investigation, Methodology, Writing – original draft. YZ: Data curation, Investigation, Writing – original draft. KZ: Methodology, Writing – original draft. ZB: Methodology, Writing – original draft. AL: Formal Analysis, Writing – original draft. CL: Conceptualization, Funding acquisition, Validation, Writing – review & editing. LL: Conceptualization, Funding acquisition, Validation, Writing – review & editing.
